# Predictors of Drug Retention and Survival Rate of bDMARDs in Rheumatoid Arthritis: A Four-Year Real-Life Tunisian Experience

**DOI:** 10.31138/mjr.090723.pof

**Published:** 2024-01-31

**Authors:** Soumaya Boussaid, Houssem Tbini, Sonia Rekik, Saadaoui Khaled, Safa Rahmouni, Khaoula Zouaoui, Salem Riahi, Hela Sahli, Mohamed Elleuch

**Affiliations:** 1 Rheumatology Department, La Rabta Hospital, Tunis, Tunisia; 2 Faculty of Medicine of Tunis, University Tunis el Manar, Tunis, Tunisia; 3 Resaerch Unit LR 05 SP 01, La Rabta Hospital, Tunis, Tunisia; 4 National Health Insurance Fund of Tunisia

**Keywords:** rheumatoid arthritis, biologic drugs, retention, survival rate, bDMARDs

## Abstract

**Introduction:**

This study aims to investigate the efficacy and tolerance of biologic disease-modifying anti-rheumatic drug (bDMARDs) in the current management of rheumatoid arthritis (RA) by identifying the retention time and survival rate of bDMARDs.

**Materials and Methods:**

We conducted a retrospective cohort study including Tunisian patients initiating bDMARD treatment between 2016 and 2018 whose data were collected from the National Health Insurance Fund (NHIF). The NHIF is the national office which organises and centralises patients under bDMARDs from all over the country. Retention and survival rate of bDMARDs at 48 months were analysed using Kaplan-Meier survival curves and compared using the log-rank test. Survival factor analysis was performed using Cox regression.

**Results:**

Three hundred seventy-four patients, aged 55.5±12.5years [20–90], (87.2%women), were included. The mean duration of RA was 11.7±6.7 years [2–41]. The mean disease activity score (DAS)28 at initiation of the first bDMARD was 6.01±0.89 [5.37–6.5]. This first bDMARD induced low disease activity (LDA) in 55% of cases. Remission was observed in 28% of patients. The highest LDA and remission rates were observed with Tocilizumab (70.8% and 33.3% of cases, respectively). LDA and remission were achieved within a mean of 45 weeks [26–88] and 72 weeks [31–117] respectively. The 48-month first-line survival rate was 55.9%. Retention time was 41.7 months, 95%CI [39.47–43.91]. Presence of rheumatoid factors, co-prescription of methotrexate, and good initial therapeutic response were factors influencing better survival of bDMARDs (p<0.01). Glucocorticoid use predicted poorer survival (p<10-3). The first bDMARD was interrupted in 39% of cases. Ineffectiveness was the most common cause of treatment cessation (52.7%).

**Conclusion:**

This real-life study of the Tunisian population allowed us to establish the factors that can influence the survival and retention rates of bDMARDs.

## INTRODUCTION

The emergence of biologic disease-modifying anti-rheumatic drugs (bDMARDs) in the late 1990s revolutionised the management of rheumatoid arthritis (RA).^1^ These therapies can control symptoms, reduce inflammation, limit joint destruction, induce clinical remission, prevent functional impairment^2^ and thereby reduce the morbidity and mortality associated with RA.^3^ Despite the evidence for the efficacy and safety of bDMARDs from several randomised therapeutic trials, the validity of these studies in real life is significantly reduced due to inclusion and exclusion criteria.^4^ For these reasons, national registries have been established to study the efficacy and safety of bDMARDs in a real-world setting. The retention time and survival rate of bDMARDs varies considerably depending on several factors that deserve clarification.

Indeed, the survival of a drug can be considered an indicator of its efficacy, safety, and tolerability. The latter two are the most common reasons for discontinuation of bDMARDs,^5^ however, drug survival can also be influenced by other factors, mainly related to patient characteristics.^6,7^

As many factors affect the survival of bDMARDs, the results of international studies could not be extrapolated to our population. Therefore, we conducted this study to determine i) the retention time and survival rate of bDMARDs in the Tunisian population, ii) the predictive factors of bDMARDs failure or discontinuation.

## MATERIALS AND METHODS

### Data Source

This was a retrospective cohort study (conducted from 2016 to 2020) of bDMARDs claims made for 374 Tunisian patients at the National Health Insurance Fund (NHIF) (office that centralises all patients on bDMARDs, in order to operate them nationwide).

The study protocol was reviewed and approved by our local ethics committee. Informed consent was obtained.

### Cohort Selection

We included RA patients over 18 years old, in whom the diagnosis of RA (code 17M05 of the NHIF) was established according to the ACR/EULAR2010 criteria and who started the first line bDMARD between January 2016 and December 2018. The index date was defined as the date of the first intake of this bDMARD. The identified bDMARDs were those with a marketing authorisation in Tunisia during this period: TNF inhibitors (TNFi): Infliximab (IFX), Adalimumab (ADA), Etanercept (ETN), and Certolizumab Pegol (CZP); IL6 receptor inhibitor (IL6Ri): Tocilizumab (TCZ); and CD20 inhibitor (CD20i): Rituximab (RTX). The choice of bDMARD is made according to international and national recommendations and contraindications to the prescription of any of the available bDMARDs. In case of inefficiency or side effects, the treatment is changed to another class or molecule.

Unusable records and those of patients with bDMARDs agreement without follow-up were excluded. Patients without regular follow-up, non-adherent to treatment and whose doses of conventional disease-modifying anti-rheumatic drug (csDMARDs) and/or corticosteroids were modified were not included.

### Outcome

The main outcome was bDMARDs efficacy and tolerance by specifying their survival rate and retention time. The second outcome was the failure or drop-out of bDMARDs by specifying factors influencing treatment survival rate.

### Study Parameters

Retention time for bDMARDs has been defined as the time from initiation of bDMARDs to discontinuation, which may be due to ineffectiveness, adverse effects, or other reasons.^8^ bDMARDs drug survival at a given time point is the rate of patients who retained the same bDMARD at this time.

### Data Gathering

The included records were analysed. Missing data were collected by telephone interviews with patients.

We collected epidemiological data of the study population: age, gender, comorbidities; and RA characteristics: disease duration, presence of rheumatoid factor (RF), anti-citrullinated peptide antibodies (ACPA) and antinuclear antibodies (ANA), and extra-articular manifestations. Structural lesions were not specified because of the lack of data necessary for evaluation by a validated score such as the Sharp score.

We have also detailed the treatments used in these patients: symptomatic treatment including corticosteroids, csDMARDs [methotrexate (MTX), sulfasalazine (SLZ), synthetic antimalarial (SA), leflunomide (LEF)], and bDMARDs.

The time from index date to discontinuation (or study end date) was calculated for each patient and defined as the drug retention time. We also specified the time needed to reach low disease activity (LDA) or remission, and the time needed to increase bDMARDs injection/infusion intervals.

RTX discontinuation was considered when the interruption lasted more than 1 year.

RA activity was assessed using the Disease Activity Score 28 (DAS28). Follow-up at 3 months after the index date and then every 3 or 6 months, depending on disease activity, was performed in all patients. Assessment of therapeutic response was based on the change in DAS28 (delta DAS28) according to the EULAR response criteria.^9^

### Statistical Analysis

Data were analysed using IBM SPSS Statistics v.26 software. We used frequencies, means±standard deviation, or medians for the study of the population characteristics. Survival information was studied by constructing survival curves using the Kaplan Meier method. Prognostic factors for survival were studied in univariate analysis (factor by factor) by comparing survival curves with the log-rank test. The risk factors associated with each event were identified via multivariate analysis using descending stepwise Cox regression. The significance level was set at a p-value<0.05.

## RESULTS

The study included 374 patients, of which 77.8% (n=291) were younger than 65 years. RF and ACPA data were reported in 324 (86%) and 267 (71%) patient records, respectively.

ETA was the most prescribed TNFi (54%) followed by ADA (14%), CZP (13%), and IFX (6%). The clinical, biological, and therapeutic characteristics of patients are presented in **Table 1**.

**Table 1.** Clinical, biological, and therapeutic characteristics of patients at bDMARDs initiation.

**Characteristics**		**Value**
**Age** (mean±SD [min-max]) years		55±12.54 [20–90]
**Male/female (n (%))**		48 (12.8)/326 (87.2)
**Disease duration** (mean±SD [min–max]) years		11.7±6.76 [2–41]
**FR+** (n (%))		296 (79)
**ACPA+** (n (%))		270 (72)
**Sjögren’s syndrome** (n (%))		23 (6.14)
**Pulmonary involvement** (n (%))		19 (5.08)
**Rheumatoid nodule** (n (%))		5 (1.33)
**Hip involvement** (n (%))		24 (4.01)
**Atlantoaxial dislocation** (n (%))		15 (4)
**DAS28** (mean±SD [min–max])		6.02±0.82 [5.37–6.5]
**Methotrexate** (n (%))		365 (97.6)
**Methotrexate dose** (mean±SD [min–max]) mg/week		19±2.84 [10–25]
**Methotrexate+Sulfasalazine** (n (%))		219 (58.6)
**Methotrexate+Leflunomide** (n (%))		147 (31.3)
**Methotrexate+Synthetic antimalarial** (n (%))		83 (22.2)
**Corticosteroids** (n (%))		214 (57.2)
**Mean dose corticosteroids** (mean±SD [min–max]) (mg/day)		6.20±2.34 [2.5–20]
**TNFi** (n (%))		323 (86.4)
	**IFX** (n (%))	23 (6.1)
	**ADA** (n (%))	52 (13.9)
	**ETN** (n (%))	202 (54)
	**CZ**P (n (%))	46 (12.3)
**Methotrexate+TNFi** (n (%))		223 (59.6)
	**Methotrexate dose** (mean±SD [min–max]) mg/week	15.1±4.2 [7.5–25]
	**Oral/intramuscular route** ((n (%)/n (%))	185 (83)/38 (17)
**TCZ** (n (%))		24 (6.4)
**RTX** (n (%))		27 (7.2)

SD: standard deviation; min: minimum; max: maximum; FR+: positive rheumatoid factor; ACPA+: positive anti-citrullinated protein antibodies; DAS: disease activity score; TNFi: TNF inhibitor; IFX: Infliximab; ADA: Adalimumab; ETN: Etanercept; CZP: Certolizumab Pegol; TCZ: Tocilizumab; RTX: Rituximab.

### Response to the First bDMARD

At 3 months, the response was good in 77.2% of cases and moderate in 11.1% of cases. However, 11.7% of patients had not responded. ETN was associated with the highest response rate (77.8%).

The first bDMARD induced LDA in 55% of cases. Remission occurred in 28% of patients.

The highest LDA and remission rates were observed with Tocilizumab (70.8% and 33.3% of cases, respectively).

The mean time to LDA and remission was 45 weeks [26–88] and 72 weeks [31–117], respectively.

The survival rate of the first bDMARD was 55.9% at 4 years of treatment. The first bDMARD was retained for a mean duration of 41.7 months, 95%CI [39.47–43.91].

The time to LDA and remission, drug survival rates and retention time for each bDMARD are detailed in **Table 2**.

**Table 2.** Response to the first-line bDMARDs.

	**Time to LDA (weeks) [min–max]**	**Time to remission (weeks) [min–max]**	**Drug survival (%)**				**Drug retention (months) [CI 95%]**
			12 months	24 months	36 months	48 months	
**ETA**	70.60 [8.7–220.1]	89.50 [8.7–199.9]	87.13	70.30	60.04	48.40	41.78 [38.78–44.78]
**ADA**	38.20 [10.1–78.7]	57.17 [10.5–113.6]	86.27	66.60	53.64	48.40	37.51 [31.86–43.15]
**CTZ**	44.00 [11.1–107.6]	46.70 [11.1–120.6]	87.5	76.75	71.64	-	30.96 [27.82–34.11]
**IFX**	61.50 [18.3–145.4]	94.80 [29.7–160]	81.82	54.17	49.24	28.14	32.3 [24.83–39.24]
**TCZ**	49.90 [8.9–106.6]	78.90 [26.9–171.9]	95.83	83.33	77.38	–	40.07 [35.24–44.89]
**RTX**	30.10 [17.4–42.9]	28.20 [17.4–42.9]	66.67	62.75	58.26	58.26	37.57 [30.05–45.08]

LDA: Low disease activity; min: minimum; max: maximum; CI: confidence interval; TNFi: TNF inhibitor; ETA: Etanercept; ADA: Adalimumab; CTZ: Certolizumab; IFX: Infliximab; TCZ: Tocilizumab; RTX: Rituximab.

bDMARDs intervals were increased in 100 patients (27%) due to sustained remission. This interval increase involved, in most cases, patients on TNFi. The first interval increase occurred after a mean time of 104.6±46.4 weeks [52–162.3]. With a mean DAS28 before interval increase of 2.8±0.8 [2.1–3.2] and after interval increase of 2.9±0.57 [2.5–3.4]. The mean difference in DAS28 of interval increase was 0.86±0.2 (p=0.364). IFX doses were optimised to 5mg/kg in three patients without efficacy. The first bDMARD was discontinued in 39% of cases for various reasons **(Table 3)**. Switching to a second treatment was made in 79 patients (21.1%). Fifty-four patients received TNFi, 19 received TCZ, and 6 received RTX. CZP was the most prescribed second-line bDMARD (41.8%).

**Table 3.** Causes of bDMARDs discontinuation.

**Number of patients (n=146)**		
**Inefficiency**	Primary	32
	Secondary	45
**Intolerance**	Cutaneous	8
	Respiratory	4
	Hepatic	2
	Digestive	1
**Neoplasm/Haemopathy**	Breast cancer	3
	Adenocarcinoma of the upper rectum	1
	Bladder carcinoma	1
**Severe Infection**	Intestinal amoebiasis	1
	Peritonitis	1
	Tuberculosis	1
	Fungal sepsis	1
**Other**	Patient lost to follow-up	13
	Remission/LDA not obtained	12
	Not justified	9
	Lack of social security coverage	5
	Death	3
	Social fund decision	2
	Lupus	1

### Factors Affecting First-Line bDMARD Retention Time and Survival Rate

We assessed drug survival based on therapeutic response three months after the index date and found a statistically significant difference between “no response,” “moderate response,” and “good response” (p<10-3) with a higher rate for patients with moderate and good response.

Regarding disease activity, we found that the higher the delta DAS28 at 3 months of treatment, the lower the risk of discontinuing bDMARDs, as reflected by the Hazard Ratio (HR:0.709, 95%CI [0.321–0.937], p<10-3).

Age did not influence bDMARDs survival (HR=0.997, 95%CI [0.763–1.122], p=0.667).

Male gender appeared to be a predictor of bDMARDs discontinuation (HR=1.442, 95%CI [1.252–1.941]), however this result was not statistically significant (p=0.107).

RF positivity was predictive of better survival of bDMARDs (HR=0.643, 95%CI [0.321–0.845], p=0.023). This was not similar for ACPA positivity (HR=1.004, 95%CI [0.982–1.123], p=0.987).

The combination of RA and Sjögren’s syndrome could increase the risk of discontinuation of bDMARDs (HR=1.236). However, this risk was not significant (p=0.519).

The combination of MTX with bDMARDs reduced the risk of discontinuation (HR=0.506, p<10-3) in contrast to corticosteroid therapy, which significantly increased this risk (HR=1.781, 95%CI [0.531–1.982], p=0.001).

The various clinical, immunological, and therapeutic factors and their influence on the survival and retention time of bDMARDs are detailed in **Tables 4 and 5**.

**Table 4.** Factors influencing the bDMARDs survival rate.

		**Survival rate at 12 months (%)**	**Survival rate at 24 months (%)**	**Survival rate at 36 months (%)**	**Survival rate at 48 months (%)**	**p**
**Age**	<65 years	87.29	70.33	59.46	54.41	0.521
	≥65 years	81.71	69.54	65.30	60.94	
**Gender**	Female	86.50	72.04	62.11	56.62	0.102
	Male	81.25	55.80	51.05	51.05	
**Immunological status**	RF -	86.76	63.17	42.79	35.50	**0.020**
	RF +	85.16	69.90	61.91	58.80	
	ACPA -	90.67	69.26	58.01	52.05	0.986
	ACPA +	84.37	67.13	58.02	49.21	
**Sjogren’s Syndrome**	SS -	85.75	70.85	61.05	56.13	0.514
	SS +	86.96	56.52	56.52	56.52	
**RA treatment**	MTX -	81.46	59.52	47.38	42.89	**<10^3^**
	MTX +	88.79	77.04	69.71	64.90	
	Corticosteroid therapy -	87.50	76.76	70.65	68.10	**<10^3^**
	Corticosteroid therapy +	84.58	64.90	53.04	45.96	
	Nonresponse to bDMARDs	71.79	51.28	39.89	28.49	**<10^3^**
	Moderate and good response to bDMARDs	92.83	76.41	66.45	63.87	

RF: Rheumatoid Factor; ACPA: Anti-Citrullinated Protein Antibodies; SS: Sjögren’s syndrome; RA: Rheumatoid Arthritis; MTX: Methotrexate; bDMARDs: biologic disease modifying antirheumatic drugs.

**Table 5.** Drug retention time, according to clinical, immunologic, and therapeutic features.

		**Mean duration (months)**	**CI 95%**
**Age**	<65 years	41.43	[38.93–43.93]
	≥65 years	42.21	[37.51–46.92]
**Gender**	male	42.3	[39.98–44.46]
	female	36.31	[30.08–42.53]
**Immunological status**	RF -	34.97	[30.06–39.89]
	RF +	42.15	[39.43–44.87]
	ACPA -	40.05	[35.30–44.80]
	ACPA +	40.05	[36.87–43.24]
**Sjogren’s Syndrome**	SS +	41.83	[39.54–44.12]
	SS -	39.83	[31.26–48.40]
**RA treatment**	MTX -	34.86	[31.55–38.17]
	MTX +	45.46	[42.74–48.18]
	Corticosteroid therapy -	45.34	[42.25–48.43]
	Corticosteroid therapy +	38.32	[35.31–41.33]
	Nonresponse to bDMARDs	28.55	[22.41–34.69]
	Moderate and good response to bDMARDs	45.35	[43.06–47.64]

CI: Confidence interval; RF: Rheumatoid Factor; ACPA: Anti-Citrullinated Protein Antibodies; SS: Sjögren’s syndrome; RA: Rheumatoid Arthritis; MTX: Methotrexate; bDMARDs: biologic disease modifying antirheumatic drugs.

**Figure 1.**
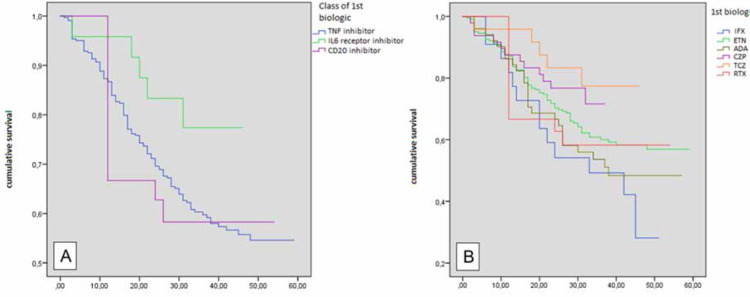
Survival curves according to (A): the class of the first bDMARD; (B): each bDMARD separately.

Impact of bDMARDS on their Survival Rate and Retention Time We found no significant difference between the survival curves according to the class of the bDMARD (TNFi, IL6Ri and CD20i) (p=0.208). Similarly, when studying the curves of all bDMARDs separately (IFX, ADA, ETN, CZP, TCZ and RTX), no difference was detected between survival curves (p=0.109) **(Figure 1)**. Compared to IFX, the risk of discontinuation was statistically lower for CZP (HR=0.443, p=0.037) and TCZ (HR=0.304, p=0.024), respectively.

### Multivariate Analysis

Multivariate analysis concluded that the combination of corticosteroids with bDMARDs doubles the risk of bDMARD failure (HR=2.127, p<10-3).

In addition, RF positivity is associated with better drug survival (HR=0.558, p=0.006). Similarly, the combination with MTX was associated with a reduced risk of bDMARDs discontinuation of up to 48% (HR=0.522, p<10-3).

Moreover, therapeutic response at 3 months was a key factor in bDMARDs survival. A moderate or good initial response was associated with better bDMARDs survival by reducing the risk of discontinuation by 66% (HR=0.434, p<10-3).

In contrast, female gender and IFX were associated with an increased risk of discontinuation (HR=1.186, HR=1.682, respectively).

## DISCUSSION

This study of RA patients on bDMARDs showed an average response to first-line bDMARDs after four years of follow-up. Indeed, LDA was observed in half of our patients and remission was achieved in about a quarter. The four-year first-line survival rate was 55.9% and the bDMARD was maintained for approximately four years. The most effective bDMARD was TCZ. Among TNFi, the highest rate of good response was observed under ETN (77.83%). RF positivity, MTX combination, and good initial therapeutic response were factors influencing the survival of bDMARDs. Corticosteroids use predicted a lower survival rate.

### Factors Influencing Survival and Therapeutic Retention

#### Demographic factors

Some studies had not found an association between age and treatment retention,^10–12^ which is consistent with our results, in contrast to Seung Min et al. who found that elderly RA patients were more likely to discontinue bDMARDs within 24 months.^13^ On the other hand, Jino S et al. had studied Japanese patients with elderly-onset RA treated with TNFi and CTZ and found that drug retention was significantly higher with CTZ. Moreover, discontinuation due to lack of efficacy was significantly less frequent in CTZ while discontinuations due to adverse event or achievement of clinical remission were similar between the two groups.^14^

The mean duration of RA was 11.7 years. The literature data were divergent on this subject. Indeed, the different screening and treatment strategies could induce this disparity in the disease duration. In addition, the RA duration induced poor therapeutic response, especially for bDMARDs and was a risk factor for discontinuation, much more for reasons of side effects than inefficacy.^15,16^ These patients are more sensitive to the properties of these immunosuppressive molecules and are at higher risk of side effects.

#### Immunological biomarkers

There is conflicting opinion regarding the impact of RF and ACPA on survival of bDMARDs.^17^ Lv et al.^18^ concluded that immunological status did not influence the response to TNFi. However, Ogawa et al. noted that RF positivity was associated with a higher rate of TNFi discontinuation, whereas ACPA had no effect on drug survival. This is explained by the fact that IgM has a greater potential to activate complement, causing greater inflammation, which may explain the more frequent cessation of TNFi in the presence of IgM isotype RF. However, ACPA, being IgG, are associated with less discontinuation.^17,19,20^

On the other hand, in line with our results, Ching-Tsai Lin et al. found that IgM isotype RF positivity was negatively associated with drug survival (HR=0.48, 95%CI[0.27–0.85], p=0.013).^21^ Mulligen et al. also agreed with these results.^22^ This could be explained by the fact that RF- and ACPA-negative RA generally show less bone erosion, structural damage, and disease progression.^23^ Thus, the presence of these autoantibodies could be associated with a more aggressive disease responding less to bDMARDs.

This discrepancy could be due in part to the different genetic and ethnic characteristics of populations, the definition of the outcomes, and statistical methods.

#### Disease activity

The mean DAS28 on initiation of bDMARDs was 6.02±0.82 [5.37–6.5] in our study and between 3.95 and 6.3 in the literature.^24,25^

The initial DAS28 was an indicator of survival of bDMARDs, as suggested by Hetland et al.^26^ and Gabay et al.^27^ who had shown that a high DAS28 at initiation of bDMARDs allowed better retention. These results were not in agreement with the Spanish study by Leon et al. who showed that a DAS28 score>5.^1^ was not associated with subsequent survival of bDMARDs.^28^

We did not evaluate the association of initial DAS^28^ with survival rates in our study. In fact, all patients had an initial DAS28>5.^1^.

This was the condition for granting bDMARDs via NHIF.

#### RA Treatments

Concomitant use of corticosteroids was observed 57.2% in our study and between 43.5 and 75% in the literature.^29–31^ Its impact on the survival of bDMARDs has been studied by several authors who had identified an increased risk of discontinuation.^16,32,33^ Souto et al.^33^ explained these findings by having more severe RA requiring corticosteroid use with a greater risk of treatment failure and discontinuation. These results were consistent with our results. However, no effect of corticosteroids on the efficacy and safety of bDMARDs was found in a study that pooled data from four double-blind randomised controlled trials in RA (AMBITION, ACT-RAY, AD-ACTA, and FUNCTION).^34^

Although EULAR recommendations suggest the combination of TNFi with MTX in RA,^35^ up to one-third of patients with RA are treated with TNFi as monotherapy.^36^ MTX-TNFi combination was prescribed in 59.6% of our patients. These results were lower than those of Kobayakawa et al.^30^ Ebina et al.^29^ and Ramiro et al.^31^ who reported this combination in 75, 68.6 and 69% of cases, respectively. This disparity could be explained by a lower adherence to the oral route due to poor tolerance. This combination was predictive of better survival of bDMARDs in our study and other studies.^5,15,29,33,37^

#### Choice of bDMARDs

To date, there are no validated criteria for selecting the first-line bDMARD. This depends on comorbidities, systemic manifestations, and complications, as well as national and international recommendations.^38–40^ Several studies had not shown significant differences between these products in terms of clinical, functional or structural efficacy.^41–44^ TNFi was the most commonly prescribed first-line bDMARD.^41,42^

Among the TNFi in our study, CZP allowed a faster achievement of LDA (44 weeks [11.1–107.6]) and remission (46.7 weeks [11.1–120.6]) compared to the others. This was in agreement the PREDICT, RAPID-1, and RAPID-2 studies, which showed that CZP reduced disease activity and improved functional indices, fatigue, and pain in patients from the 12th week of treatment.^45–47^ More interestingly, Nakayama et al. had shown that RA patients with high serum RF (>166IU/ml) treated with TNFi without Fc fragment had significantly lower DAS28-ESR at 12 months of treatment compared with those treated with TNFi with Fc.^48^

Furthermore, among all bDMARDs in our study, TCZ had the highest LDA (70.8%) and remission rate (33.3%).

Our remission rate was lower than those in the literature (ranging from 45 to 86%).^49–51^ This could be explained by a longer duration of RA and the low percentage of men in our cohort. Indeed, remission is less frequent in women than in men.^52^

In addition, the retention time of the first bDMARD was 41.7 months [39.4–43.9]. This result was close to those reported by Ramiro et al. (49.2 months) and Brodszky et al. (42.8 months).^31,53^ Furthermore, the ANSWER cohort study showed that Abatacept and TCZ had higher retention, and TCZ had lower rates of discontinuation due to inefficacy compared with IFX. In contrast, IFX had a higher rate of discontinuation for remission than Abatacept, ETN, Golimumab, and TCZ in adjusted modeling.^29^

Hishitani et al.^54^ had found a retention time for TCZ, IFX, ETN and ADA of 2.5, 1.9, 2.9, and 1.3 years, respectively.

The authors of the Danish DANBIO registry,^55^ concluded that the median survival rate was 45 months for ETA and 29 months for IFX and found low remission rates despite long drug survival. However, Soubrier et al. found that there was no difference in retention between ADA, ETA, and IFX. Twenty-five percent of patients continued treatment for 15 years.^32^

Furthermore, in a recent systematic literature review including 170 publications, the survival rate of ETN was higher than that of the other TNFi after more than one year of follow-up.^56^ On the other hand, in a cohort study by Choi S et al. patients who received first-line TCZ, Tofacitinib or abatacept had a higher survival rate compared to first-line ETN.^57^ These discrepancies in retention time and survival rates could be explained by variations in patient recruitment methods, disease duration, activity at baseline, changes in global assessment, etc.^58^

Initial therapeutic response may predict better bDMARDs survival rates. Wei et al.^59^ had shown that the survival rate is better for patients who had initially responded well to treatment. Indeed, as the progression index (CDAI) decreases, the therapeutic survival improves. This was in agreement with our study.

To our knowledge, this study is one of the few to address the retention and survival of bDMARDs in an Arab and African population. It investigates the real-life survival of bDMARDs and highlights their value in the treatment of RA. Identification of factors that may affect survival would allow optimisation of treatment outcomes by acting on modifiable factors among them. However, our study has some limitations, mainly because of its open-label design, which is more prone to bias compared with double-blind controlled trials. In addition, the cohort size was relatively limited. Moreover, because of the “real-life” study design, the continued use of other medications, such as glucocorticoids and MTX, might affect the results. Another limitation of this study is that the data were collected from the NHIF database; recruitment bias may exist in the selection of patients to receive bDMARDs. Furthermore, given the significant disparity in the number of patients on different classes of bDMARDs and the fact that most of our patients were on TNFi (86.4%). The conclusions drawn from our comparative study remain to be validated by other, larger studies with similar proportions of bDMARDs classes.

## CONCLUSION

The overall management of RA has improved significantly in recent decades thanks to bDMARDs. These treatments, not devoid of risk and not always effective, may be discontinued for one reason or another, hence the interest in studying their survival. The literature on the subject was divergent. Our study identified factors influencing biological survival, including RF positivity, MTX-TNFi combination, corticosteroid use and good initial response to treatment. Action on modifiable factors could improve bDMARDs survival, enabling better disease control. Larger-scale African and Arab studies would be needed to support our results.

## Data Availability

The datasets used and/or analysed during the current study are available from the corresponding author on reasonable request.
